# Phenotypic Screening of the MMV Global Health Priority Box Identifies Selective Compounds with Anti-*Toxoplasma gondii* Activity

**DOI:** 10.1021/acsomega.5c05130

**Published:** 2025-07-10

**Authors:** Gabriel Candido Moura, Juliana Quero Reimão

**Affiliations:** 146840 Faculdade de Medicina de Jundiaí, Jundiaí 13202-550, São Paulo, Brazil

## Abstract

Toxoplasmosis remains a globally significant parasitic disease, with limited treatment options and reports of drug intolerance and inadequate efficacy, particularly against chronic stages. This study aimed to identify novel anti-*Toxoplasma gondii* compounds through the phenotypic screening of the Medicines for Malaria Venture (MMV) Global Health Priority Box (GHPB), a curated library of 240 chemically diverse molecules. Parasite viability and host cell cytotoxicity assays identified six lead compounds with the selectivity index (SI) exceeding 100, with MMV689404 (Triflumuron), MMV1794214 (Vaniliprole), and MMV1794211 showing SI >103, > 756, and >1000, respectively. *In silico* ADMET profiling revealed favorable pharmacokinetic and toxicity parameters for most hits, although Vaniliprole showed suboptimal gastrointestinal absorption and potential tumorigenicity. Comparative analysis with the existing literature confirmed previously reported antiparasitic activity for several compounds, reinforcing their relevance for repurposing. These findings highlight the potential of GHPB as a source of candidate molecules for further preclinical evaluation against toxoplasmosis.

## Graphical abstract

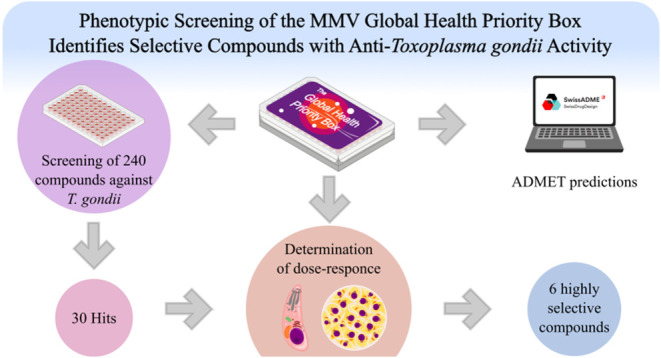

## Introduction

Toxoplasmosis is a globally prevalent zoonotic infection caused by the protozoan parasite *Toxoplasma gondii*, affecting nearly one-third of the world’s population.^1^ While most immunocompetent individuals experience asymptomatic or mild disease, the parasite poses serious health risks to immunocompromised individuals—such as patients with AIDS or undergoing immunosuppressive therapies—and to pregnant women, in whom congenital transmission can result in miscarriage, stillbirth, or severe neurological sequelae in the fetus.^2^ Despite the global burden and clinical relevance, therapeutic options for toxoplasmosis have remained largely unchanged for decades.

Current first-line treatments, typically based on the combination of pyrimethamine and sulfadiazine, are associated with significant drawbacks, including bone marrow suppression, hypersensitivity reactions, and poor activity against the latent bradyzoite stage. Moreover, growing concerns over drug tolerance and suboptimal parasite clearance emphasize the urgent need for new, safer, and more effective treatment alternatives.^3^

In this context, drug repurposing has emerged as a promising approach, offering the advantage of accelerating therapeutic discovery by evaluating compounds with known safety and pharmacological profiles. One particularly valuable resource is the Global Health Priority Box (GHPB), assembled by the Medicines for Malaria Venture (MMV). This curated collection comprises 240 chemically diverse compounds with established bioactivity against malaria and other neglected pathogens,^4^ yet their potential against *T. gondii* remains unexplored.

In this study, we conducted a phenotypic screening of the GHPB library to identify compounds with anti-*T. gondii* activity. Our aim was to prioritize candidates with favorable selectivity indices and pharmacokinetic properties for further preclinical development, contributing to the discovery of novel chemotherapeutic options for toxoplasmosis.

## Results

A primary screening of 240 compounds from the Global Health Priority Box (GHPB) library was performed at a fixed concentration of 1 μM with a 72 h incubation period. Compounds were considered active if they inhibited *T. gondii* growth by ≥80% under these conditions.

This screening identified 30 active compounds: 6 from the VEC subset, 10 from MB2, and 14 from ZND (Figure 1). These hits were selected for further characterization, including the determination of their EC_50_ in intracellular *T. gondii* tachyzoites, CC_50_ in human foreskin fibroblasts (HFF), and selectivity index (SI = CC_50_/EC_50_). Pyrimethamine, a standard drug used to treat toxoplasmosis, was included as a reference.

**Figure 1:**
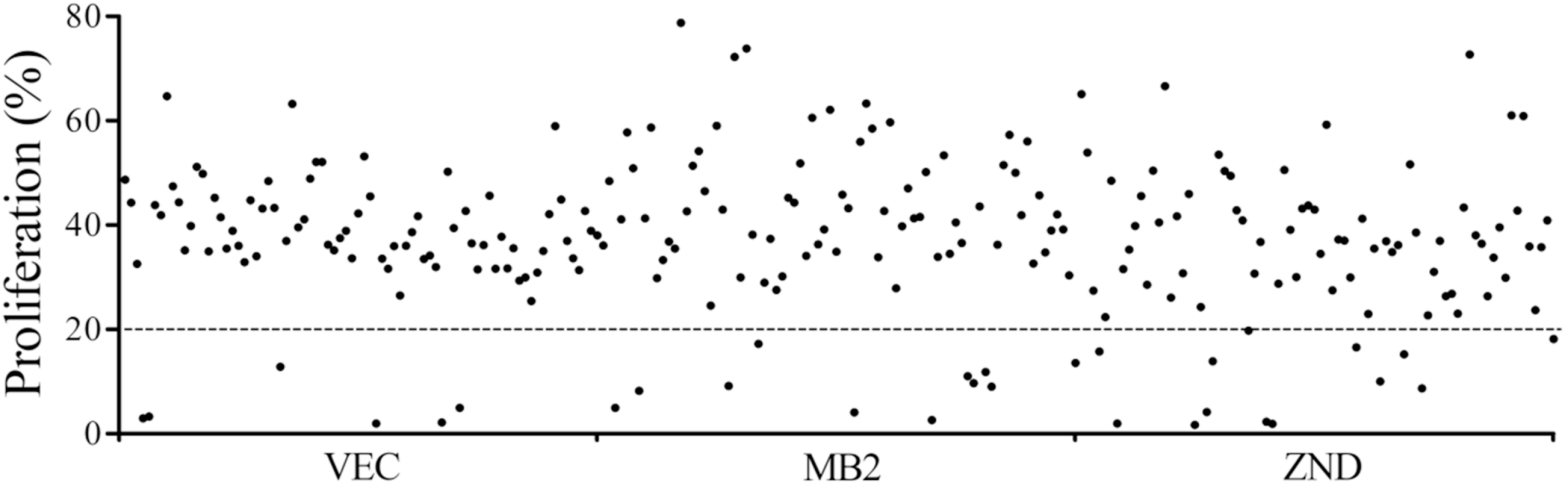
Screening of 240 compounds of the GHPB collection in fixed 1 μM concentration against *T. gondii* proliferation.

### Potency and Selectivity

EC_50_ values of the selected compounds ranged from 0.01 to 0.90 μM, indicating a wide spectrum of anti-*T. gondii* potency. CC_50_ values ranged from 0.38 to >50 μM, the highest concentration tested. Selectivity indices varied considerably among the compounds, from <1 to >1000 (Table 1).

**Table 1:** EC_50_, CC_50_, and SI of the GHPB Compounds^a^

GHPB plate	compound ID	EC_50_ (μM) ± SD	CC_50_ (μM) ± SD	SI
VEC	MMV1794214	0.07 ± 0.04	>50	>756
	MMV1794211	0.01 ± 0.01	>10^b^	>1000
	MMV689404	0.49 ± 0.03	>50	>103
	MMV027339	0.50 ± 0.02	5.51 ± 0.86	11
	MMV1577471	0.51 ± 0.01	4.62 ± 1.95	9
	MMV1634081	0.52 ± 0.01	30.83 ± 4.55	59
MB2	MMV1841740	0.90 ± 0.12	6.47 ± 0.76	7
	MMV006187	0.51 ± 0.12	>50	>98
	MMV006430	0.30 ± 0.03	>50	>167
	MMV024638	0.50 ± 0.04	4.98 ± 0.90	10
	MMV674132	0.19 ± 0.04	>50	>265
	MMV1267536	0.19 ± 0.03	29.20 ± 2.79	154
	MMV1266067	0.73 ± 0.13	>50	>68
	MMV019421	0.62 ± 0.15	30.39 ± 10.32	49
	MMV1435700	0.34 ± 0.08	20.00 ± 3.80	59
	MMV024825	0.62 ± 0.08	22.24 ± 0.17	36
	MMV1103183	0.72 ± 0.39	>50	>70
ZND	MMV1795508	0.59 ± 0.15	6.80 ± 0.46	11
	MMV692115	0.56 ± 0.08	3.38 ± 0.06	6
	MMV1593278	0.21 ± 0.09	6.36 ± 0.22	30
	MMV1828776	0.24 ± 0.13	2.77 ± 0.78	11
	MMV1828058	0.10 ± 0.08	2.43 ± 0.2	25
	MMV1828026	0.74 ± 0.12	1.33 ± 0.36	2
	MMV884814	0.76 ± 0.06	4.10 ± 1.18	5
	MMV1545678	0.81 ± 0.15	6.47 ± 0.14	8
	MMV692630	0.72 ± 0.03	10.99 ± 0.54	15
	MMV689635	0.61 ± 0.04	>50	>81
	MMV689463	0.83 ± 0.21	2.66 ± 0.95	3
	MMV1848726	0.63 ± 0.12	0.38 ± 0.26	0.6
	MMV688723	0.73 ± 0.09	>50	>68
external control	Pyrimethamine	0.38 ± 0.03	>50	>131

a EC_50_: Half effective concentration against *T. gondii* intracellular tachyzoites; CC_50_: Half cytotoxic concentration against HFF; SI: Selectivity index; SD: Standard deviation. Results obtained from duplicates in three independent experiments.

b According to Tali et al. in VERO/RAW cells.^5^

Six compounds showed particularly high selectivity (SI > 100): MMV1794211 (SI > 1,000), MMV1794214 (SI > 756), MMV674132 (SI > 265), MMV1267536 (SI = 154), MMV006430 (SI > 167), and MMV689404 (SI > 103). The chemical structures of these compounds are shown in Figure 2.

**Figure 2:**
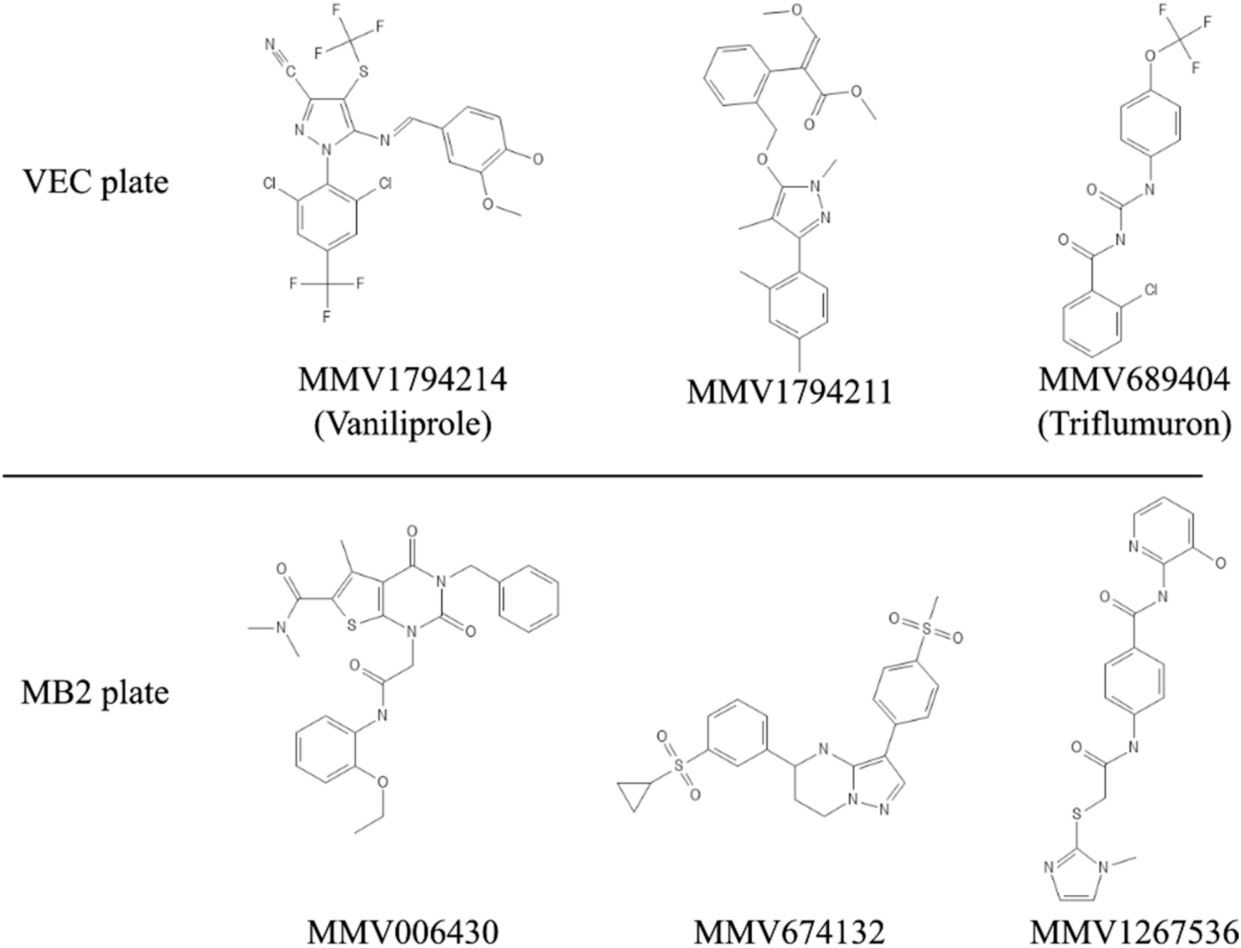
Chemical structure of compounds with SI > 100.

On the other hand, several compounds such as MMV1828026 and MMV1848726 presented low SI values (2 and 0.6, respectively), indicating high toxicity relative to antiparasitic activity. Pyrimethamine showed an EC_50_ of 0.38 μM and an SI > 131, serving as a useful benchmark.

### ADMET Profile

To assess drug-likeness and predict potential safety issues, the 30 active compounds were subjected to *in silico* ADMET profiling, including predictions of gastrointestinal (GI) absorption, blood–brain barrier (BBB) permeability, and toxicity risks (Table 2).

**Table 2:** Results of ADMET Predictions of the Selected Compounds from GHPB^a^ ^,^ ^b^

	compound ID	molecular formula	GI	BBB	mutagenic risk	tumorigenic risk	irritant risk	reproductive effect risk
VEC	MMV1794214	C_20_H_10_Cl_2_F_6_N_4_O_2_S	low	no	no	medium	no	no
	MMV1794211	C_25_H_28_N_2_O_4_	high	yes	no	high	medium	high
	MMV689404	C_15_H_10_ClF_3_N_2_O_3_	high	no	no	no	no	no
	MMV027339	C_15_H_8_Cl_2_F_6_N_2_O	low	no	no	no	no	no
	MMV1577471	C_25_H_24_F_6_N_4_	low	no	no	no	no	no
	MMV1634081	C_13_H_11_Cl_2_N_3_O4	high	no	no	no	no	high
MB2	MMV1841740	C_20_H_18_F_3_N_5_	high	no	no	no	no	no
	MMV006187	C_24_H_20_FN_3_OS	low	no	no	no	no	no
	MMV006430	C_27_H_28_N_4_O_5_S	high	no	no	no	no	No
	MMV024638	C_23_H_20_FN_3_	high	yes	no	no	no	no
	MMV674132	C_22_H_23_N_3_O_4_S_2_	high	no	no	no	no	no
	MMV1267536	C_18_H_17_N_5_O_3_S	high	no	no	no	no	no
	MMV1266067	C_16_H_14_N_4_O_2_	high	no	no	no	no	no
	MMV019421	C_26_H_28_ClN_5_O_2_S	high	no	no	no	high	no
	MMV1435700	C_18_H_17_N_3_O_4_S_2_	low	no	no	no	no	no
	MMV024825	C_25_H_26_F_2_N_4_O	high	yes	no	no	no	no
	MMV1103183	C_15_H_16_N_4_O_3_	high	no	no	no	no	no
ZND	MMV1795508	C_17_H_15_ClF_3_N_5_	high	no	no	no	no	no
	MMV692115	C_21_H_21_Cl_2_N_5_O	high	yes	no	no	no	no
	MMV1593278	C_18_H_25_FN_4_	high	yes	no	no	no	no
	MMV1828776	C_23_H_25_N_5_O	high	yes	no	no	no	no
	MMV1828058	C_26_H_30_FN_5_O	high	no	no	no	no	no
	MMV1828026	C_26_H_26_N_6_O_2_	high	no	high	high	no	no
	MMV884814	C_24_H_23_ClF_3_N_5_O	high	no	no	no	no	no
	MMV1545678	C_21_H_24_Cl_2_N_6_	high	no	no	no	no	no
	MMV692630	C_21_H_16_ClF_3_N_4_O_2_	high	no	no	no	no	no
	MMV689635	C_23_H_19_ClN_6_O_2_	high	no	no	no	no	no
	MMV689463	C_23_H_24_ClN_5_O	high	yes	no	no	medium	no
	MMV1848726	C_19_H_17_N_5_O_2_	high	no	no	no	no	no
	MMV688723	C_21_H_19_ClN_4_O_3_S	high	no	no	no	no	no
	pyrimethamine	C_12_H_13_ClN_4_	high	yes	high	high	no	high

a GI: gastrointestinal absorption.

b BBB: blood–brain barrier permeability.

Most compounds (25/30) were predicted to have high GI absorption. Five compounds, MMV1794214, MMV027339, MMV1577471, MMV006187, and MMV1435700, showed low predicted GI absorption, which may impact oral bioavailability.

Only seven compounds were predicted to cross the BBB, including MMV1794211, MMV024638, MMV024825, MMV692115, MMV1593278, MMV1828776, and MMV689463. This may be advantageous for the potential treatment of cerebral toxoplasmosis.

Most compounds showed low predicted toxicity, but six were flagged for potential safety concerns: MMV1794211, MMV1794214, MMV1634081, MMV019421, MMV1828026, and MMV689463.

Interestingly, despite the highlighted risks in some candidates, pyrimethamine itself was predicted to have high mutagenic, tumorigenic, and reproductive toxicity risks, underscoring the unmet need for safer alternatives.

To better visualize the relationship between compound potency and selectivity, a scatter plot was generated comparing EC_50_ and SI for the six most selective compounds (SI > 100). Each compound was color-coded according to its predicted ADMET-related toxicological risks, including mutagenicity, tumorigenicity, irritancy, and reproductive effects. This visualization (Figure 3) highlights the most promising candidates based on their pharmacological profiles and simultaneously flags compounds with potential safety concerns. Notably, several compounds with SI > 100 also exhibit favorable ADMET predictions (e.g., MMV674132 and MMV1267536), whereas others, such as Vaniliprole, despite high SI, raise concerns due to predicted tumorigenic risk.

**Figure 3:**
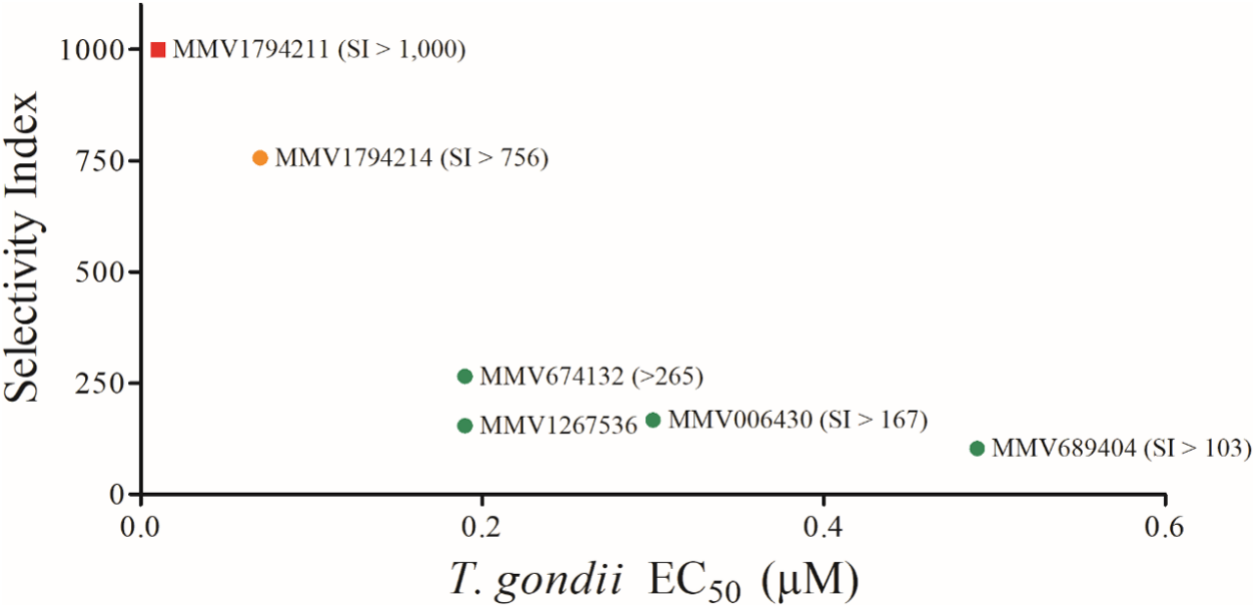
Correlation between anti-*T. gondii* *in vitro* activity (EC_50_) and the selectivity index (SI) for the six most selective compounds (SI > 100). Colors indicate predicted toxicological risks: green = no major risks; orange = moderate risk in at least one category; and red = high or multiple predicted risks. Square indicates the predicted blood–brain barrier (BBB) permeability, while circles indicate no predicted BBB permeability.

## Discussion

Previous screenings of the GHPB library have demonstrated bioactivity against fungi, including *Candida auris* and *Madurella mycetomatis*;^7,6^ protozoa such as *Plasmodium falciparum*, *Plasmodium berghei*, *Leishmania amazonensis*, *Trypanosoma cruzi*, and *Naegleria fowleri*;^13–9^ helminths such as *Hemonchus contortus;* ^11^ and SARS-CoV-2.^12^ Nevertheless, its efficacy against *T. gondii* remains unexplored, presenting a critical gap in the discovery of novel therapies targeting toxoplasmosis.

Screening of the GHPB library identified 30 promising compounds that were subjected to additional *in vitro* assays. To contextualize these findings, we analyzed compound *in vitro* activity using EC_50_ against the selectivity index (SI) of the most selective compounds (SI > 100). Among these, Vaniliprole (MMV1794214) stood out with an SI > 756 and potent activity (EC_50_ = 0.07 μM), but its predicted ADMET profile suggests potential tumorigenic effects and low gastrointestinal (GI) absorption, reducing its suitability as a drug lead despite promising *in vitro* data.

A particularly notable finding emerged with MMV1794211, for which cytotoxicity could not be determined under our experimental conditions, even after repeated assays. Given this limitation, we adopted the CC_50_ value >10 μM reported by Tali et al.,^5^ which, combined with its observed antiparasitic activity (EC_50_ = 0.01 μM), results in a calculated SI > 1000. This extraordinarily high selectivity places MMV1794211 among the top candidates in the library. Despite the absence of reliable in-house cytotoxicity data, its lack of predicted ADMET toxicological risks, coupled with its exceptional SI, strongly supports its prioritization for further evaluation. Future studies will be necessary to clarify its safety profile and determine its mechanism of action.

In contrast, compounds MMV006430, MMV674132, and MMV1267536 from the MB2 plate combine high SI values (>167, >265, and 154, respectively) with favorable ADMET predictions (high GI absorption, no toxicity alerts), making them strong candidates for further development. Notably, MMV674132 and MMV1267536 have also shown activity against *Plasmodium* liver stages,^13^ indicating potential for dual antiparasitic applications. In addition, MMV006430 and MMV674132 have been experimentally characterized as fast-acting compounds against *P. falciparum* ring stages, showing activity under 65 h.^10^

Our findings for Vaniliprole (MMV1794214) align with previous reports of its antiparasitic potential. Shanley et al. (2024)^11^ demonstrated that this compound impairs motility and larval development in *H. contortus*, highlighting its anthelmintic efficacy. In our study, Vaniliprole also showed potent activity against *T. gondii* (EC_50_ = 0.07 μM) and an exceptionally high SI (>756), supporting its broad antiparasitic potential.

Similarly, our data for Triflumuron (MMV689404) corroborate some aspects of its known pharmacological profile. We observed an SI > 100 and favorable *in silico* ADMET parameters, including high GI absorption and low toxicity prediction. However, Timoumi et al.^14^ reported CC_50_ values of 120 μM and 200 μM in HepG2 and HEK293 cell lines, respectively, and subsequent *in vivo* studies showed hepatotoxicity and oxidative stress at doses of 350–500 mg/kg (Timoumi et al.,).^15^ These toxicological findings, which contrast with our predictive data and *in vitro* results, underscore the importance of integrating *in vivo* toxicity data early in the candidate selection process, particularly for compounds with prior regulatory or pesticidal usage.

While no compounds from the ZND plate exceeded SI > 100, this may reflect methodological limitations, as CC_50_ values were capped at 50 μM, potentially underestimating actual selectivity. Indeed, several ZND compounds cluster near the high-potency, moderate-selectivity region, suggesting that they warrant further cytotoxicity profiling using expanded concentration ranges.

The integrated analysis reveals that selectivity alone is insufficient for prioritization; compounds must also exhibit an acceptable predicted safety. This is exemplified by MMV1828026, a ZND compound with reasonable potency (EC_50_ = 0.74 μM) but a predicted high mutagenic and tumorigenic risk, rendering it unsuitable despite initial interest.

In summary, the visual synthesis of potency, selectivity, and safety predictions enabled the rational prioritization of lead-like compounds. MMV1794211, MMV674132, and MMV1267536 emerge as especially promising based on their combined potency, selectivity, and predicted safety profiles, warranting further evaluation through mechanism-of-action studies and *in vivo* models.

## Conclusions

This study screened compounds from the GHPB library and identified several candidates with potent anti-*T. gondii* activity and favorable selectivity profiles. Notably, six compounds (MMV1794211, MMV1794214, MMV674132, MMV1267536, MMV006430, and MMV689404) exhibited SI values greater than 100. While Vaniliprole and Triflumuron stood out for their strong efficacy, their translational potential is limited by ADMET concerns and previously reported *in vivo* toxicity. In contrast, compounds from the MB2 plate, such as MMV674132 and MMV1267536, combined promising efficacy with more favorable *in silico* safety profiles, warranting further investigation. Collectively, our findings underscore the potential of the GHPB library as a valuable resource for the discovery of new anti-*T. gondii* agents and lay the groundwork for future studies aimed at confirming efficacy, elucidating mechanisms of action, and evaluating *in vivo* efficacy and safety.

## Methods

### Drugs and Chemicals

All chemicals and reagents were obtained from Thermo Fisher Scientific (Leicestershire, U.K.). The Global Health Priority Box (GHPB) library (240 compounds) was generously provided by the Medicines for Malaria Venture (MMV, Geneva, Switzerland). The library consists of three distinct 80-compound subsets: the first plate is notable for containing compounds with reported activity against various disease vectors (VEC), the second plate comprises drugs demonstrating confirmed efficacy against resistant malaria (MB2), while the third plate contains a library of compounds previously screened against neglected zoonotic diseases (ZND).^4^ Stock solutions (10 mM in DMSO) were prepared under sterile conditions and stored at – 20 °C.

### Parasite and Host Cell Culture

*Toxoplasma gondii* RH-2F1 tachyzoites, which express β-galactosidase, were maintained in human foreskin fibroblasts (HFFs). HFFs were cultured in Dulbecco’s Modified Eagle Medium (DMEM) supplemented with 10% fetal bovine serum (FBS), 2 mM l-glutamine, and 40 μg/mL gentamicin (referred to as D10) and incubated at 37 °C in a 5% CO_2_ humidified atmosphere. For infection assays, confluent monolayers were maintained in DMEM with 2% FBS (D2) prior to parasite inoculation.^16^

For *in vitro* assays in 96-well plates, HFFs were seeded at 5 × 10^3^ cells/well in the D10 medium and incubated for 12–16 h to allow adherence. For cytotoxicity (CC_50_) evaluation, cells were treated with test compounds in serial dilutions ranging from 50 to 0.78 μM for 72 h. For antiparasitic assays, HFFs were infected with RH-2F1 tachyzoites at a multiplicity of infection (MOI) of 1:1 for 3 h. After infection, compounds were added either at a fixed concentration (1 μM for screening) or in serial dilutions (1 to 0.008 μM for EC_50_ determination), followed by 72 h incubation under standard conditions.

### β-Galactosidase Quantification

Parasite proliferation was assessed by quantifying β-galactosidase activity, as previously described.^10^ Briefly, after the 72 h incubation, cells were lysed in 100 μL of lysis buffer (100 mM HEPES pH 7.5, 1 mM MgSO_4_, 0.1% Triton X-100, 5 mM DTT) for 15 min at room temperature. Subsequently, 160 μL of assay buffer (100 mM sodium phosphate, pH 7.3, 102 mM β-mercaptoethanol, 9 mM MgCl_2_) and 40 μL of CPRG substrate (6.25 mM) were added to each well. Following a 30 min incubation at 37 °C, absorbance was measured at 570 nm using a Varioskan LUX microplate reader (Thermo Scientific). Each assay included viability controls (untreated infected cells), solvent controls (1% DMSO), a positive control (1 μM pyrimethamine), and experimental blanks (no cells). All conditions were tested in two biological replicates in three independent experiments.

### GHPB Library Screening

Compounds from the GHPB library were screened at a final concentration of 1 μM in infected HFF monolayers, using the β-galactosidase assay as a readout for parasite viability after 72 h. Compounds exhibiting ≥80% inhibition of parasite proliferation were selected for further evaluation in dose–response assays to determine the EC_50_ and CC_50_ values.

### Cytotoxicity Assay (Resazurin Reduction)

The resazurin assay was used to evaluate the compound cytotoxicity in uninfected HFFs. Following 72 h treatment, 100 μM resazurin was added to each well and incubated for 4 h at 37 °C. Fluorescence was measured using excitation/emission wavelengths of 565/590 nm. Controls included untreated cells (viability control), 1% DMSO (solvent control), 50 μM pyrimethamine (reference drug), and blank wells (no cells). Each condition was tested in duplicate in three independent experiments.

### *In Silico* ADMET Analysis

ADMET (absorption, distribution, metabolism, excretion, and toxicity) properties of selected hits were predicted using SwissADME (http://www.swissadme.ch)^17^ and OSIRIS Property Explorer.^18^ The evaluated parameters included gastrointestinal (GI) absorption, blood–brain barrier (BBB) permeability, and predicted toxicity (mutagenicity, tumorigenicity, irritation, and reproductive effects).

## Abbreviations

GHPB: global health priority box
MMV: medicines for malaria venture
VEC: various disease vectors plate
MB2: resistant malaria plate
ZND: neglected zoonotic diseases plate
HFF: human foreskin fibroblast
DMEM: Dulbecco’s modified eagle medium
FBS: fetal bovine serum
CC_50_: half cytotoxicity concentration
EC_50_: half effective concentration
MOI: multiplicity of infection
ADMET: absorption, distribution, metabolism, excretion and toxicity
GI: gastrointestinal
BBB: blood–brain barrier
SI: selectivity index
